# The impact of the COVID-19 pandemic on oral health inequalities and access to oral healthcare in England

**DOI:** 10.1038/s41415-021-3718-0

**Published:** 2022-01-28

**Authors:** Michelle Stennett, Georgios Tsakos

**Affiliations:** Department of Epidemiology and Public Health, University College London, 1-19 Torrington Place, London, WC1E 7HB, UK

## Abstract

While the impact of the coronavirus disease (COVID-19) pandemic on health inequalities is documented, oral health has been absent from this discussion. This commentary highlights the potential impacts of the COVID-19 pandemic on oral health inequalities in England in February 2021. It includes a literature review, Public Health England and Kantar Worldpanel sales data on health behaviours and analysis of NHS dental services data. Purchasing data indicate, except for smoking, increases in health-compromising behaviours. Since the resumption of dental services, NHS general dental service use modestly recovered among adults but not children by October 2020. There are clear inequalities among children and older adults, with more deprived groups having lower uptake of dental service use than more affluent groups. Oral cancer referrals and hospital admissions for tooth extractions in children dramatically declined, with the latter primarily affecting children in more deprived areas. Many oral health programmes in schools and care homes were disrupted or suspended throughout this period. All these indicate that oral health inequalities have widened due to the COVID-19 pandemic. An oral health plan of action requires prioritising long-term investment in public health programmes and transforming commissioning pathways to support those with the greatest needs to access oral healthcare services.

**Supplementary Information:**

Zusatzmaterial online: Zu diesem Beitrag sind unter 10.1038/s41415-021-3718-0 für autorisierte Leser zusätzliche Dateien abrufbar.

## Key points

- People living in more deprived areas have fared worse than people living in less deprived areas, in terms of uptake of NHS primary dental care following the resumption of services in June 2020.
- The cessation of oral health improvement programmes in the community and the dramatic decline of hospital dental services due to the COVID-19 pandemic have also primarily impacted the more socially disadvantaged groups, further widening inequalities.
- Addressing the widened oral health inequalities requires long-term investment in oral health, prioritising public health programmes and supporting access to services.

**Supplementary Information:**

Zusatzmaterial online: Zu diesem Beitrag sind unter 10.1038/s41415-021-3718-0 für autorisierte Leser zusätzliche Dateien abrufbar.

## Introduction

Inequalities in oral health have been widely documented in the UK and are evident across the social spectrum and life course, largely reflecting the socio-economic inequalities in general health.^1^^,^^2^^,^^3^^,^^4^^,^^5^ Furthermore, marginalised and vulnerable groups (such as homeless people and care home residents) present the cliff-edge of inequality, having excessively poorer oral health outcomes, often coupled with considerable barriers and limited access to oral healthcare compared to the general population.^6^

In the last 18 months, the emergence of COVID-19 has led to unprecedented economic and public health crises globally.^7^^,^^8^ The UK has seen one of the highest mortality rates per head of population in the world^9^ and has experienced a significant economic contraction, having implications for job security, income and consequently, health.^10^^,^^11^ In addition, contrary to suggestions that COVID-19 is a 'socially neutral disease', there is clear evidence that COVID-19 has disproportionately affected socially disadvantaged groups.^12^^,^^13^ Therefore, it is postulated that the COVID-19 pandemic will have a worsening long-term effect on health inequalities in the UK, further to an existing backdrop of widening health inequalities since 2010.^14^^,^^15^

Oral health inequalities have been largely absent from this discussion. This is despite the fact that oral conditions present a global public health problem and are socially patterned and highly prevalent with a considerable burden on individuals and societies, while, at the same time, are largely preventable.^16^^,^^17^^,^^18^ This commentary aims to highlight and discuss the potential impact of the COVID-19 pandemic on oral health inequalities in England.

As there is currently no direct evidence of the impact of the COVID-19 pandemic on oral health outcomes, we looked at the potential impact of the COVID-19 pandemic on key health behaviours, as well as access to and provision of oral healthcare services, including preventive programmes, as they are expected to influence inequalities in oral health. We reviewed the literature and used Public Health England (PHE) data, Kantar Worldpanel sales data on health behaviours and NHS dental service data.

## The COVID-19 pandemic and oral health behaviours

High sugar consumption, poor oral hygiene, smoking and alcohol consumption are all known risk factors for oral diseases.^19^^,^^20^^,^^21^^,^^22^ Health-compromising behaviours cluster in lower socio-economic groups^23^^,^^24^ and present a pathway towards oral health inequalities.^25^^,^^26^ There is generally limited data on the impact of the COVID-19 pandemic on oral health behaviours. However, some relevant information can be extracted by looking at changes in purchasing patterns, though clearly this is not a direct reflection of actual behavioural practices.

### Diet

**Fig. 1:**
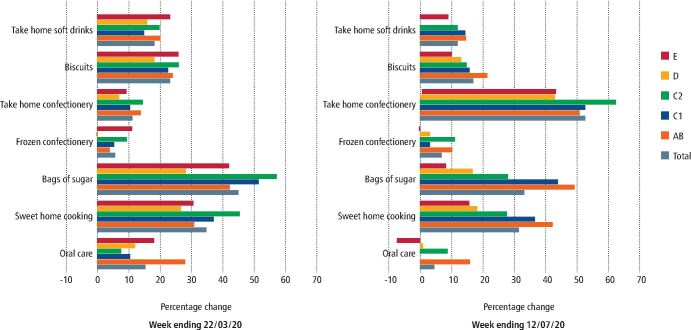
Percentage change in average volume product sales over four weeks in 2020 compared to the same weeks in 2019 (weeks ending 22 March 2020 and 12 July 2020) by social class. AB = higher and intermediate managerial, administrative and professional workers. C1 = supervisory, clerical and junior managerial, administrative and professional workers. C2 = skilled manual workers. D = semi-skilled and unskilled manual workers. E = people on long-term state benefits, casual and lowest grade workers, unemployed with state benefits (including pension) only. Data source: Public Health England. Wider impacts of COVID-19 on health analysis of Kantar Worldpanel data 2020^30^

Prior to the COVID-19 pandemic, few people met dietary recommendations and although those from higher income groups were nearer to achieving some recommendations, diets did not meet recommendations across all income groups.^27^ Compared to the corresponding weeks in 2019, there were increases in the reported purchase of confectionery, biscuits and sweet home cooking (all foods 'rich' in free sugars) among adults in the weeks before the initial lockdown (March 2020) and also later in June/July 2020 (Fig. 1). High intakes of sugars are a major risk factor for dental caries and can also lead to excess calorie consumption, thereby increasing the risk of becoming overweight or obese.^28^ Both of these are more prevalent in deprived groups.^29^

### Oral hygiene

There was some variation in oral care product purchases around the time of the initial lockdown. Compared to the corresponding weeks in 2019, there was a large increase in oral care product purchases across all social classes, but more so for the higher social classes, just before the initial lockdown in March 2020. However, the pattern changed by June/July 2020, when the increases were more modest for the higher social classes and there was even a decrease in oral product purchases for the lowest social class (Fig. 1).

### Smoking

There were no significant changes in the prevalence of smoking according to socio-economic position over the course of the initial lockdown, but increases in attempts to quit smoking and in cessation rates among smokers were noted in both managerial and skilled manual workers.^30^ There is currently not enough evidence to understand whether these changes may affect the longer-term prevalence of smoking between the different socio-economic groups and thereby potentially further influence oral health inequalities.

### Alcohol consumption

A PHE evidence review in 2016 found that the harmful effects of alcohol were greatest in deprived groups.^31^ Data suggest that alcohol sales/consumption increased overall during 2020.^32^ Kantar Worldpanel sales data analyses measuring alcohol purchasing in off-trade settings (such as supermarkets) also suggest that the volume of alcohol sales increased by 36% between March and June 2020. This may be expected, since on-trade settings (for example, pubs) were closed for those four months. However, alcohol duty receipts (which include on- and off-trade sales) indicate that people consumed more alcohol than in previous years. Surveys suggest a polarisation in self-reported drinking patterns, with similar proportions reporting drinking more and less alcohol than before the first national lockdown.^32^ Importantly, evidence suggests that the heaviest 20% of buyers of alcohol before the COVID-19 pandemic accounted for 45% of the total increase in sales following the pandemic.^32^ Put together, data from sales and surveys suggest that despite the closure of the on trade at the beginning of the COVID-19 pandemic, alcohol sales increased and this increase was driven by heavy consumers of alcohol. Consequently, this high-risk group of heavy drinkers is now, following the lockdown, even more likely to experience alcohol-related harm with implications for oral health, particularly for oral cancer.

## The COVID-19 pandemic and oral healthcare services

### Access to primary dental care

**Fig. 2:**
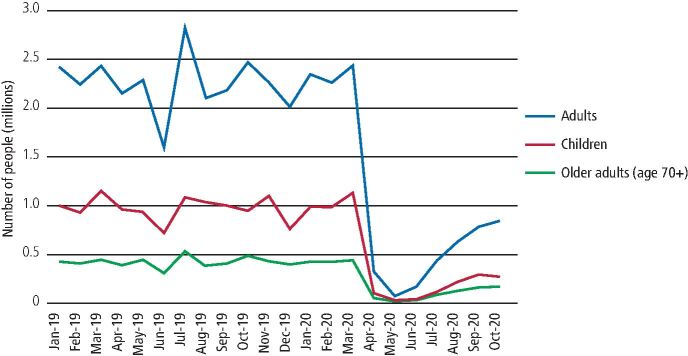
Number of people accessing general dental services in England from January 2019 to October 2020 by age group and month. Data source: NHS Business Services Authority 2020

With the initial lockdown in March 2020, all routine and non-urgent dental care ceased. Instead, urgent dental care centres were established to provide care for people who could not be managed remotely with advice, analgesia or antibiotics.^33^ NHS general dental service data for England reflect these changes and demonstrate a dramatic decline (98%) in dental services used by children, adults and older adults in this initial lockdown period (Fig. 2). With limitations in access to routine services, there were reports regarding rises in dental infections necessitating emergency hospital treatment^34^ and increased episodes of 'do it yourself' dentistry.^35^

**Fig. 3:**
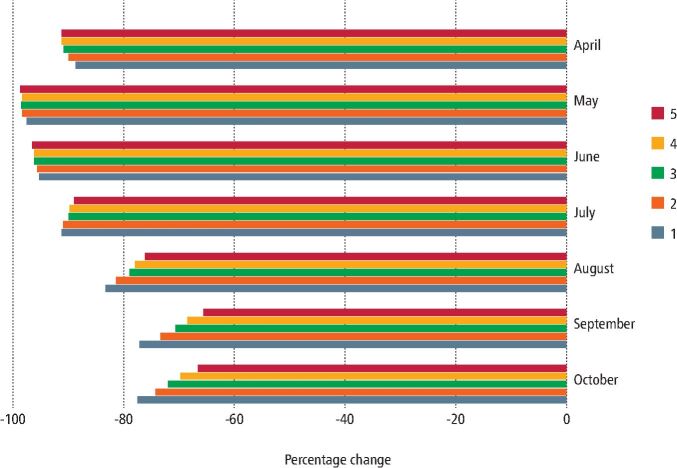
Monthly percentage change in child access to NHS dental services 2020 vs 2019 by IMD 2015 national deprivation quintile. 5 = least deprived quintile, 1 = most deprived quintile. Data source: NHS Business Services Authority 2020

In England, non-urgent dental care services resumed on 8 June 2020, though the resumption was gradual, as not all practices were ready and appropriately equipped to do so immediately. Inability to carry out aerosol generating procedures (AGPs) due to a lack of personal protective equipment and practical arrangements of care provision due to surgery down-time following AGPs affected care provision. Reports indicate continued difficulties in access to both urgent and routine dental care, with a Healthwatch report suggesting that in some mixed practices, private dental care was prioritised over NHS provision.^36^ NHS data highlighted some increase in general dental service activity following the resumption of dental services, though the activity was still considerably lower compared to the previous year, with 67% fewer people utilising services in October 2020 than in October 2019. The increase in NHS dental service activity happened more quickly and at a higher rate for adults than children (Fig. 2). Furthermore, there were clear inequalities in the uptake of dental services in this initial resumption period, particularly among children and older adults, with 10% more children and older adults in the least deprived areas of England utilising services in October 2020, compared to those in the most deprived areas (Fig. 3, online Supplementary Figures 1 and 2). These inequalities reflect NHS dental care and do not consider private provision.

### Access to secondary dental care

Tooth extractions are the most common reason for hospital admissions among children aged 6-10 years old.^37^ Along with the cessation of routine dental services in March 2020, many hospitals throughout England also cancelled elective tooth extraction lists and comprehensive care lists in order to accommodate the anticipated increased capacity needed to treat COVID-19 patients.^38^^,^^39^ The lower provision of these services also impacts people from vulnerable groups (for example, those with learning disabilities) who may require hospital services, often with general anaesthesia, for the provision of dental care.

**Fig. 4:**
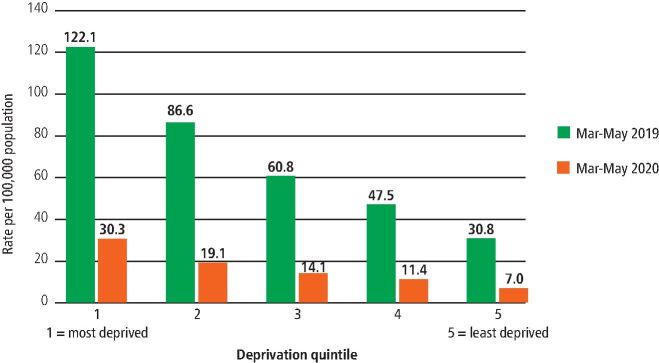
Hospital episodes of dental caries-related tooth extraction in 0-19-year-olds by deprivation quintile, rate per 100,000 population. Data source: Public Health England 2020

For oral surgery hospital referrals, 75% of patients were seen within the 18-week referral to treatment time limit in March 2020. By August 2020, this had fallen to 32% of patients.^40^^,^^41^ In 0-19-year-olds, there was a 94% decrease in hospital episodes of dental-caries-related tooth extractions in April 2020 compared with April 2019.^37^ Hospital admissions for tooth extractions are strongly socially patterned with children living in more deprived areas being more likely to receive this care and have more teeth removed during an episode.^37^^,^^38^ The reduction in hospital tooth extractions impacted, in terms of absolute numbers, more upon the deprived children, even though it was proportionately similar across socio-economic groups. For example, the rate of hospital tooth extraction episodes per 100,000 population dropped from 122 between March to May 2019 to 30 between March to May 2020 among the most deprived children (a difference of 92 per 100,000), while the respective figures among the least deprived were 31 and 7, a difference of 24 per 100,000 (Fig. 4).

Across England, re-establishment of services varied^42^ with many hospital tooth extraction services for children seeing restricted numbers of cases.^43^ In many areas, this has resulted in longer waiting lists and therefore increased treatment needs and higher impact in terms of pain, sleepless nights and missing school.^44^ The exact impact of these developments remains to be quantified, but considering the aforementioned stark inequalities, the more deprived children and vulnerable adults will be primarily affected by the continuing lower levels of service provision.

### Oral cancer services

In the UK, there are marked inequalities in rates of oral cancers, with higher incidence rates in more deprived and vulnerable groups.^45^^,^^46^ Dental examinations allow opportunistic screening for signs and symptoms of oral cancer, with early diagnosis having a profound impact on successful treatment and survival rates. With the cessation of routine dental examinations during the initial lockdown, there has been a substantial decrease in urgent referrals for suspected oral cancer.^47^^,^^48^ A reduction in referrals will have additional impacts on prompt treatment provision and referral waiting times.^49^ To put this into context, a modelling exercise predicted that a delay in oropharyngeal cancer diagnosis of up to three months can result in a 10% survival reduction over ten years.^50^

### Oral health improvement programmes

Early years and school-supervised tooth brushing programmes in England^51^ have been affected by the COVID-19 pandemic. Such programmes are commissioned to support, primarily, children with poor oral health, through an increased level of provision in more deprived areas. With the closure of all schools and early years settings (except to the children of key workers) in March 2020, oral health programmes in England were suspended. This temporary suspension continued after primary schools welcomed back children in June 2020. Revised guidance in England allowed for the restarting of these programmes in the 2020-2021 academic year, as settings and commissioners felt appropriate. The break in provision and delay in re-establishment of oral health promotion programmes has long-term implications for the oral health of young children.

On the other end of the age spectrum, vulnerable adults in care homes have been hit particularly hard by COVID-19 with excessive mortality rates.^52^ In general, oral health improvement practices in care homes have been found to be poor and routine dental care, including access to domiciliary services, is also challenging.^53^ These issues have been further highlighted by the COVID-19 pandemic. Oral healthcare services and programmes in care homes ceased as oral health professionals and health promoters had limited access to care homes for a prolonged period.^47^^,^^54^ Within this context, the inclusion of oral health in the enhanced 'Health in care homes' framework^55^ is a positive development, as dental practices can be linked to care homes through primary care networks.

## Summary and implications for action

The totality of the evidence presented above in terms of health behaviours, reduced access to services (particularly for the vulnerable) and cessation of oral health improvement programmes, indicate that the COVID-19 pandemic is likely to have a major impact on oral health and result in a widening of inequalities.

Dental care provision was dramatically reduced in the initial lockdown period and after resumption of services is still considerably lower than in the previous year, particularly among deprived children and older adults. It seems that less deprived groups are more able to navigate the changing architecture of NHS dental service provision than the more deprived. This is a serious concern, as the vulnerable, more deprived population groups have a greater reliance on the NHS for their dental care.^56^ Other important services (eg for oral cancer and secondary dental care) have also been severely affected, primarily impacting the lower socio-economic and more vulnerable groups. In addition, many oral health improvement programmes have stopped. The impacts of the COVID-19 pandemic and lockdown are considerable, not only for patients and the population, but also for the oral healthcare workforce. Clinicians and their staff have had to deal with the initial closure of routine dental services, significant changes in care delivery and accessing personal protective equipment, at the same time as caring for staff and patients and balancing family life. While this commentary reflects on the situation up to February 2021, it should be acknowledged that oral health programmes and dental services are part of an ever-changing picture as we move through the different phases of the COVID-19 pandemic.

Within this context, it is crucial to consider a plan of action to address oral health inequalities as we deal with the COVID-19 pandemic and move beyond/towards resumption of primary dental care services to pre-COVID-19 levels. It is important to re-align dental care provision and transform commissioning pathways to prioritise equity of access to oral health services. This is in line with the World Health Organisation emphasis of including oral health in the Universal Health Coverage compendium.^57^^,^^58^

However, the anticipated increased oral health burden and widened inequalities cannot be simply treated away or addressed through increased and more equitable provision of care. There is an urgent need to increase the reach of oral health improvement programmes that have evidence of effectiveness in reducing oral health inequalities, such as community water fluoridation and daily, supervised tooth brushing in early years settings.^59^^,^^60^ This highlights the need for programmes with emphasis on the more vulnerable groups - those whose 'voices' are often not heard. Addressing the broader social, environmental and commercial determinants of health and incorporating oral health within public health initiatives (for example, public health actions to reduce sugar consumption) is essential if we want to reduce oral health inequalities. This is even more of a priority in the current context of other widened inequalities due to the COVID-19 pandemic. The extensive disruption to the education of children within the UK may lead to long lasting, negative effects on educational outcomes, particularly for disadvantaged students with limited access to online resources.^61^ Moreover, the economic downturn initiated by the COVID-19 pandemic may result in decreased availability of jobs and is more likely to have greater impacts on the job prospects of people from lower socio-economic backgrounds.^61^ These impacts on the broader social determinants, while not strictly within the scope of this commentary, are all likely to have far reaching implications for widened oral health inequalities that go well beyond the observed effects of the COVID-19 pandemic on health behaviours and healthcare services.

## Conclusion

There is a need to move away from years of disinvestment in public health.^62^^,^^63^ Prioritising public health programmes and supporting equitable access to services will help to build a fairer society following the damaging impacts of the COVID-19 pandemic.^64^ After all, good oral health contributes considerably to the health and quality of life of the population.

## Supplementary Information

Supplementary Figures 1-2 (PDF 103KB)
