# Supplementary material for: The impact of the COVID-19 pandemic on oral health inequalities and access to oral healthcare in England
Source: Br Dent J. 2022 Jan 28;232(2):109–14. doi: 10.1038/s41415-021-3718-0 (PMC8796193; doi:10.1038/s41415-021-3718-0)
Supplement: Supplementary file 1 — Supplementary Figures 1-2 (PDF 103KB) [file 41415_2021_3718_MOESM1_ESM.pdf]

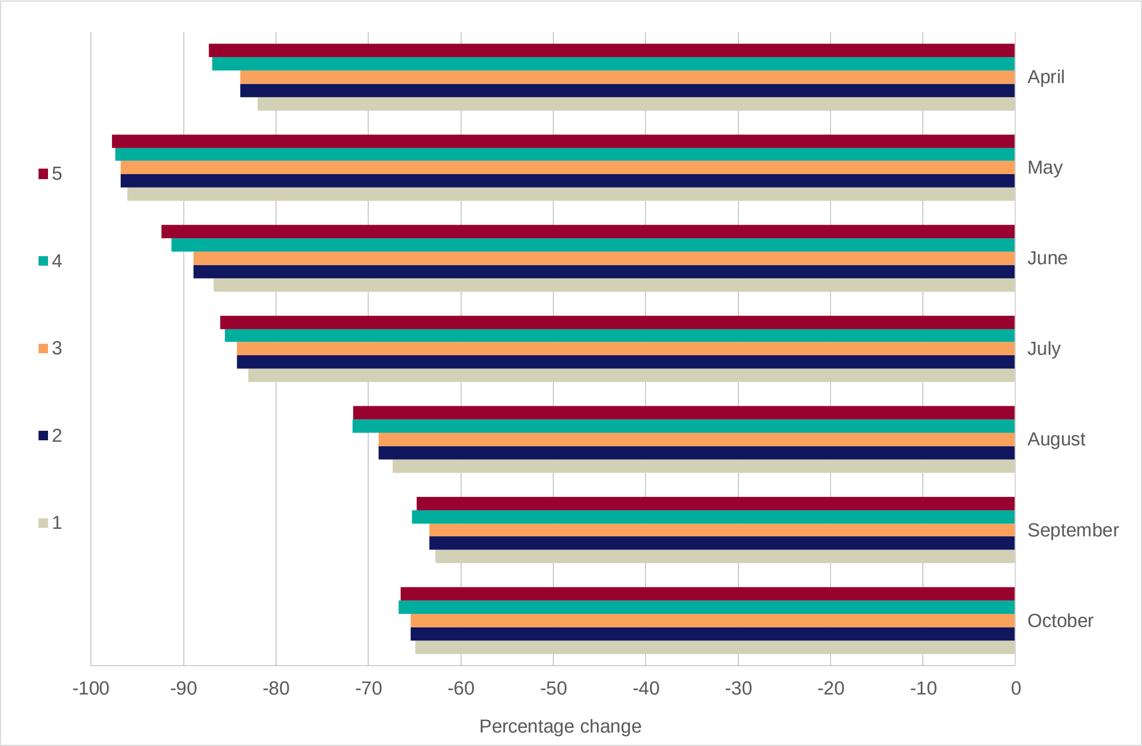

Data source: NHS Business Services Authority, 2020

Supplementary Figure 1. Monthly percentage change in adult access to NHS dental services 2020 vs 2019 by IMD 2015 national deprivation quintile
5= Least deprived quintile, 1= Most deprived quintile

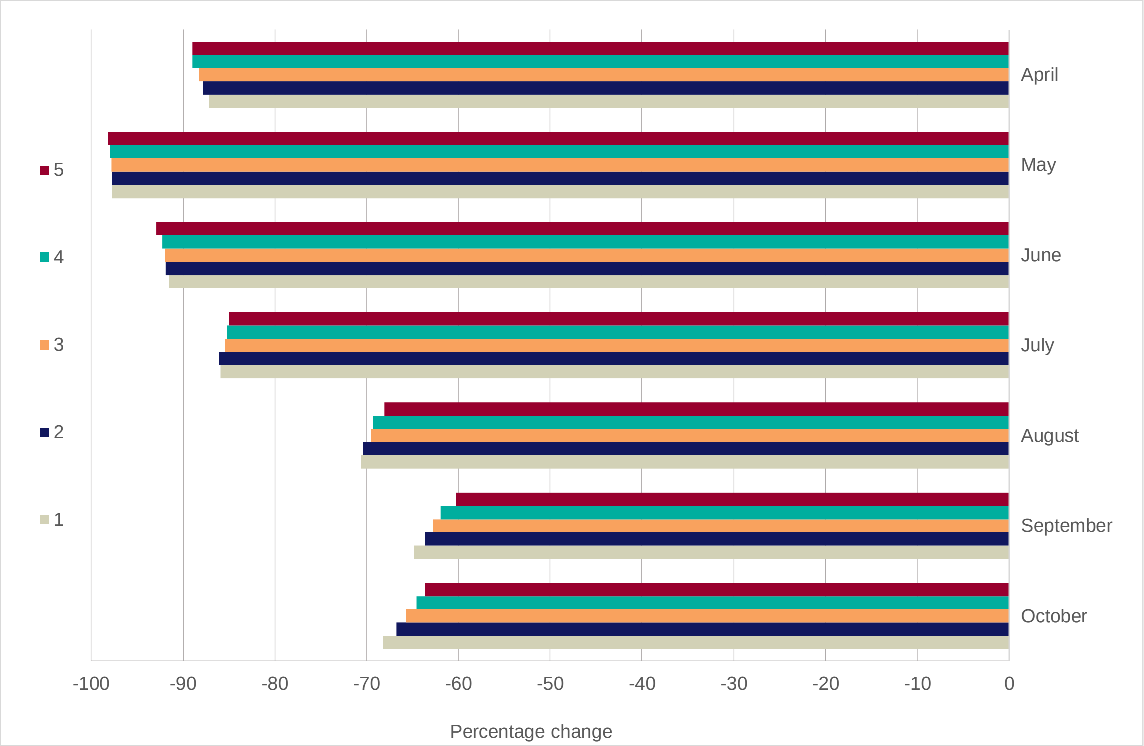

Data source: NHS Business Services Authority, 2020

Supplementary Figure 2. Monthly percentage change in older adults (aged 70 years and over) access to NHS dental services 2020 vs 2019 by IMD 2015 national deprivation quintile

5= Least deprived quintile, 1= Most deprived quintile
